# Combining tractography and cortical measures to test system-specific hypotheses in multiple sclerosis

**DOI:** 10.1177/1352458510362440

**Published:** 2010-08

**Authors:** Nikos Gorgoraptis, Claudia AM Wheeler-Kingshott, Thomas M Jenkins, Daniel R Altmann, David H Miller, Alan J Thompson, Olga Ciccarelli

**Affiliations:** 1 Department of Brain Repair and Rehabilitation, Institute of Neurology, University College London, Queen Square, London WC1N 3BG, UK.; 2 Department of Neuroinflammation, Institute of Neurology, University College London, Queen Square, London WC1N 3BG, UK.; 3 London School of Hygiene and Tropical Medicine, University of London, Keppel Street, London WC1E 7HT, UK.

**Keywords:** atrophy, MRI, relapsing–remitting multiple sclerosis

## Abstract

The objective was to test three motor system-specific hypotheses in multiple sclerosis patients: (i) corticospinal tract and primary motor cortex imaging measures differ between multiple sclerosis patients and controls; (ii) in patients, these measures correlate with disability; (iii) in patients, corticospinal tract measures correlate with measures of the ipsilateral primary motor cortex.

Eleven multiple sclerosis patients with a history of hemiparesis attributable to a lesion within the contralateral corticospinal tract, and 12 controls were studied. We used two advanced imaging techniques: (i) diffusion-based probabilistic tractography, to obtain connectivity and fractional anisotropy of the corticospinal tract; and (ii) FreeSurfer, to measure volume, thickness, surface area, and curvature of precentral and paracentral cortices. Differences in these measures between patients and controls, and relationships between each other and to clinical scores, were investigated.

Patients showed lower corticospinal tract fractional anisotropy and smaller volume and surface area of the precentral gyrus than controls. In patients, corticospinal tract connectivity and paracentral cortical volume, surface area, and curvature were lower with increasing disability; lower connectivity of the affected corticospinal tract was associated with greater surface area of the ipsilateral paracentral cortex.

Corticospinal tract connectivity and new measures of the primary motor cortex, such as surface area and curvature, reflect the underlying white and grey matter damage that contributes to disability. The correlation between lower connectivity of the affected corticospinal tract and greater surface area of the ipsilateral paracentral cortex suggests the possibility of cortical adaptation. Combining tractography and cortical measures is a useful approach in testing hypotheses which are specific to clinically relevant functional systems in multiple sclerosis, and can be applied to other neurological diseases.

## Introduction

Recent advances in MRI acquisition and analysis permit the *in vivo* investigation of pathological changes occurring in the white matter (WM) pathways and in the grey matter (GM) regions. These developments have important clinical implications in multiple sclerosis (MS), as WM and GM damage contributes to disability. One of the most useful techniques for the assessment of WM damage is diffusion-based tractography, which offers the possibility of reconstructing entire WM pathways *in vivo* and quantifying damage in neurological diseases in a tract-specific way*.*^1^ Tractography may, therefore, increase the clinical specificity of MRI in MS, relating tract damage to impairment of its associated function. Using probabilistic tractography, a voxel-based estimate of the probability of connection between two regions, called connectivity, can be obtained.^2,3^ Connectivity, which is thought to reflect the integrity of WM fibres,^4^ is an informative measure that has been used in clinical investigations,^4–6^ although less often than fractional anisotropy (FA). FA is a well established measure of axonal loss and demyelination,^7,8^ which is derived from the diffusion tensor. Although it has been shown to be lower in the tractography-derived tracts of patients with MS and amyotrophic lateral sclerosis (ALS) when compared to healthy subjects, it does not correlate with clinical scores as strongly as connectivity.^4–6,9^

Recently, evidence has been reported for extensive and clinically relevant GM damage in MS.^10,11^ The calculation of the density (or volume) of a cortical region obtained with a voxel-based morphometry (VBM) approach is most commonly performed in clinical studies, and has been interpreted as reflecting cortical atrophy.^10^ However, a reduction in GM volume can be due to thinning of the cortex, reduction in the cortical area, change of its folding, or to a combination of these processes. The FreeSurfer cortical surface-based methodology (http://surfer.nmr.mgh.harvard.edu/) allows us to estimate all these GM measures within specific brain regions in an automated way, leading to a comprehensive *in vivo* assessment of the cortical GM damage. Among these cortical measures, thickness has been investigated in a few studies in MS,^12–14^ while cortical surface area of each hemisphere of MS patients has been reported in one study only,^15^ and cortical curvature has not been investigated in MS so far.

The causes of cortical GM damage in MS are unclear.^16^ One possible mechanism is that GM axonal loss is secondary to Wallerian and retrograde axonal degeneration occurring in the WM.^17^ It is important to understand the relationship between WM and GM damage in MS, since pathology in both tissues has a clinical impact. A recent investigation of the relationship between GM volume and WM FA across the whole brain in patients with early primary progressive MS, demonstrated a link between the pathological processes occurring in both tissues.^18^ However, patients with relapsing–remitting MS (RRMS) and cortical measures were not included in this study, and the whole brain was tested without an a priori hypothesis.

Here, we focused on the motor system, investigating changes in the connectivity and FA of tractography-derived corticospinal tract (CST), and in volume, thickness, surface area, and curvature of the precentral gyrus and paracentral lobule, which are the lateral and medial part of the primary motor cortex (PMC). In order to maximize the possibility of detecting abnormalities in these regions, we studied patients with a history of hemiparesis, and a corresponding lesion in the CST. We tested the following hypotheses: (i) patients show lower FA and connectivity in the CST, and lower volume, thickness, surface area, and curvature of the PMC, when compared to controls; (ii) patients who show lower CST connectivity and lower volume, thickness, surface area, and curvature of the PMC, have greater disability; (iii) in patients, lower CST FA and connectivity correlate with lower volume, thickness, surface area, and curvature of the ipsilateral PMC.

## Methods

### Subjects

**Figure 1.**
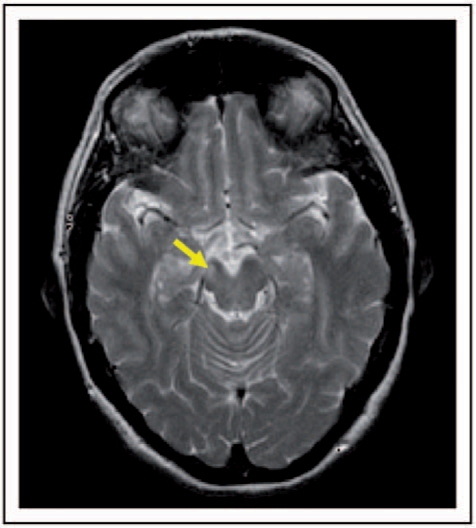
Axial T2-weighted MRI in a patient. The arrow indicates a lesion in the right corticospinal tract (CST).

We recruited patients who attended the outpatient MS clinics at the National Hospital for Neurology and Neurosurgery in London (NHNN), and fulfilled the following criteria: (i) diagnosis of MS,^19,20^ and (ii) at least 7 months history of hemiparesis, which was attributable to a lesion within the contralateral CST visible on conventional MRI (Figure 1). The 7-month interval from relapse onset ensured that patients had sufficient time to improve clinically.^21^ On the day of their scan, all patients were scored on the Expanded Disability Status Scale (EDSS),^22^ including the pyramidal functional system (FS) score, the 25-foot Timed Walk Test (TWT), and the timed 9-Hole Peg Test (9-HPT).^23^ Age- and gender-matched healthy controls were also studied.

All subjects gave written informed consent before the study, which was approved by the Joint Ethics Committee of the Institute of Neurology and The National Hospital for Neurology and Neurosurgery. All patient MRI scans were formally reported by a specialist neuroradiologist at the NHNN.

### MRI protocol

Imaging was performed on a 1.5T GE MRI scanner with an eight-channel phased array head coil and a maximum gradient strength of 33 mT/m. All subjects underwent T2-weighted fast spin-echo brain images (TR = 2.5 s, TE = 102 ms, field of view (FOV) 24 × 18 cm^2^, matrix 256 × 256, in-plane resolution 0.94 × 0.7 mm^2^, 28 contiguous axial slices, 5 mm slice thickness). T1-weighted brain images were acquired, using a 3D inversion recovery prepared spoiled gradient recall (IR-SPGR) sequence (TI = 450 ms, TR = 2 s, TE = 53 ms, FOV 310 × 155 mm^2^, matrix 256 × 128, voxel resolution 1.2 × 1.2 × 1.2 mm^3^, 156 contiguous axial slices).

Diffusion tensor imaging (DTI) data were acquired using a single-shot, diffusion-weighted (DW) echo planar imaging sequence [FOV 220 × 220 mm^2^, matrix 96 × 96 reconstructed as 128 × 128, in-plane resolution 2.3 × 2.3 mm^2^ reconstructed to 1.7 × 1.7 mm^2^, 60 contiguous axial slices, 2.3 mm slice thickness, cardiac gating (TR = 20RR ≈ 20 s), diffusion gradients applied along 61 optimized directions with a maximum *b* factor of 1200 s/mm^2^, 7 *b* ≈ 0 s/mm^2^ images].^24^

### MRI processing

#### Diffusion tensor imaging and tractography analyses

All the following steps were done using tools from the FMRIB Software Library (FSL; www.fmrib.ox.ac.uk/fsl) and applying default parameters, unless otherwise specified. Non-brain structures were removed from DW and T1-weighted images.^25^ The individual DW images were corrected for eddy current distortions and movement artefacts, and the DT was fitted on a voxel-by-voxel basis. FA maps, and all the information necessary to run probabilistic tractography, were obtained.

**Figure 2.**
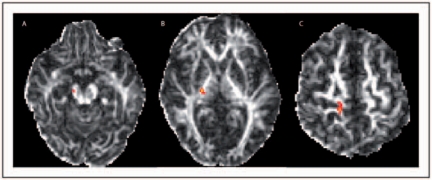
Axial fractional anisotropy images of the same patient as in Figure 1 that show the tractography-derived corticospinal tract (CST) on the right side of the brain. **A:** CST in the cerebral peduncle (*z* = 22), **B:** in the posterior limb of the internal capsule (*z* = 30), and **C:** adjacent to the right PMC (*z* = 49).

The aim of our tractography analysis was to calculate (i) the mean voxel-based connectivity, and (ii) the mean FA of the tractography-derived CST, in each subject. We used a probabilistic tractography algorithm^2^ to track the CST, from the cerebral peduncles to the PMC, and obtain a voxel-based connectivity map. Our analysis, which has been reported in detail elsewhere,^5^ included the following steps:

- Definition of cerebral peduncle and cortical areas masks. To ensure that the masks of cerebral peduncles and cortical regions were drawn in the same way in all subjects, the individual T1 images were registered into a standard space (Montreal Neurological Institute, MNI152), using affine transformations.^26^ For each side, a mask of the cerebral peduncle was designed on the standard brain, on the lowest slice where the whole cerebral peduncle was visible (*z* = 29). On the same standard brain, the PMC on the lateral and medial part of the hemisphere was drawn for each side of the brain using the MRIcro Brodmann’s atlas as a guide (http://sph.sc.edu/comd/rorden/mricro.html). A mask of the remainder of the cortex (i.e excluding the PMC) was created for each side. The masks of the cerebral peduncle and the cortical areas were then transferred back to the individual T1 images, and their correct location was confirmed visually in all cases. Probabilistic tissue type segmentation and partial volume estimation were then performed on the individual T1 images.^27^ The output images were used to mask the cortical regions to obtain the final cortical masks (i.e. the right and left PMC, and the remainder of the right and left cortex). Affine registration was then performed in each subject to transform the individual T1 images into the averaged seven *b_0_* volumes. The transformation parameters were then applied to the previously generated masks of the cerebral peduncles and of the cortex. The correct location of these masks in diffusion space was visually confirmed in all subjects.
- Connectivity-based segmentation of the cerebral peduncles*.*^5,28,29^ From each voxel in the mask of the cerebral peduncle, we drew samples from the connectivity distribution to each cortical mask. The probability of connection to a cortical mask was obtained from the proportion of samples that reached each of the cortical masks. Therefore, two classes of voxels in the cerebral peduncle were classified: those with higher probability of connection to the PMC, which constituted the ‘seed’ region in the next step, and those with higher probability of connection to the remainder of the cortex.
- Tracking the CST. Using the probabilistic tractography algorithm, we delineated the CST in each side, from the seed region to the PMC. The tractography algorithm drew 5000 samples from each voxel in the seed region to the PMC, producing a probabilistic map of the CST (Figure 2). Within this map, each voxel had a connectivity value, which corresponded to the number of samples that had passed from the seed region, through this voxel, to the target region. A threshold of 50 was applied, in line with previous work,^5,30^ and the mean connectivity of the supra-threshold voxels was calculated. The thresholded connectivity map was then binarized and used to mask the FA map, to obtain the mean value of FA within the CST, for each side, in each subject.

#### Measures of the primary motor cortex

**Figure 3.**
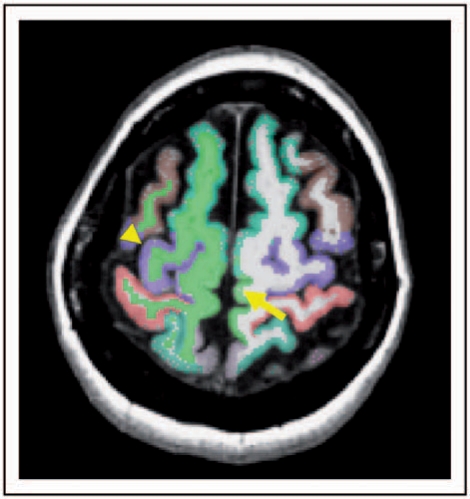
Results of the cortical parcellation overlaid onto the T1-weighted scans of the same patient as in Figures 1 and 2. The arrowhead indicates the R precentral cortex and the arrow indicates the L paracentral cortex (*z* = 58).

Cortical reconstruction and volumetric segmentation was performed with the FreeSurfer image analysis suite (http://surfer.nmr.mgh.harvard.edu/). The technical details of these procedures have been described previously.^31–39^ A fully automated parcellation of the cerebral cortex into units based on gyral and sulcal structure was performed (Figure 3).^36,40^ From this analysis, the volume, thickness, surface area, and curvature of the precentral gyrus and paracentral lobule were automatically obtained. The somatotopic, motor representation of the different body parts in the PMC, from which the majority of CST fibres originate, includes the face, hand, arm, and trunk in the lateral part of the precentral gyrus, and the lower limb in its medial part (i.e. the paracentral cortex).^41,42^

### Statistical analysis

#### Differences between the affected and unaffected side

In patients, the Wilcoxon signed rank test was used to investigate the differences in the tractography-derived CST (i.e. connectivity and FA) and in the GM measures (i.e. volume, thickness, surface area, and curvature), between the side affected by the lesion and the unaffected side. The same test was used to test for differences in the imaging measures between the left and right sides in the control group.

#### Differences between groups and association with disability

As no differences in imaging measures were found between the affected and unaffected side (see results section for details), we computed the mean of the two sides for each MRI measure, and entered it in the next analysis step. This reduced the number of comparisons and simplified the interpretation of the findings. The Mann–Whitney *U*-test was performed to assess differences in connectivity, FA, cortical thickness, and curvature between patients and controls. Since the volume and surface area variables were normally distributed, an independent samples t-test was used to compare them between the two groups. In patients, associations between all the MRI measures for each side and the clinical scores (EDSS, pyramidal FS score, TWT, and 9-HPT) were investigated using the Spearman's rank correlation coefficient (two-tailed).

#### Relationship between white matter and grey matter measures

In patients, we assessed the relationships between connectivity and FA of the tractography-derived CST and volume, thickness, surface area, and curvature of the precentral and paracentral cortices for each side using the Spearman’s rank correlation coefficient (two-tailed). The results were confirmed with bootstrap analysis.

All the analysis was performed using SPSS 15.0 (SPSS, Inc., Chicago, Illinois, USA) except bootstrap analysis which was done using Stata 9.2 (http://www.stata.com/) (StataCorp LP, College Station, Texas, USA). A *p-*value of ≤0.05 was chosen to denote statistical significance.

## Results

### Subjects’ characteristics

The patients’ clinical and radiological characteristics are reported in Table 1. Of the 11 patients recruited, seven patients had a chronic lesion in the left CST and four in the right CST (Figure 1). The location of the lesion responsible for the hemiparesis was considered to be the CST within the brain as five patients had history of facial involvement at the onset of the limb weakness, and in the remaining six patients, radiological findings provided evidence; in particular, out of these six patients, one showed a gadolinium enhancing lesion in the CST at the symptom onset, two showed a reduction in lesion size at 1 and 6 months follow-up, respectively, which was concomitant with clinical improvement, and three had cervical cord MRI at symptom onset which did not show any MS lesion. The contralateral brain CSTs did not show a T2 lesion in any patient, and none of the lesions had features suggestive of Wallerian degeneration.^43^

**Table 1.** Patients’ characteristics

Age	Mean: 46 years (SD: 13.2)
Gender	5 female, 6 male
Disease type	10 relapsing–remitting MS, 1 secondary progressive MS
EDSS	Median 4.5 (range 2–6)
Pyramidal FS score	Median 3 (range 1–4)
25-foot Timed Walk Test (TWT)	Mean 8.23 s (SD: 2.15)
9-Hole Peg Test (9-HPT)	Mean 25.4 s (SD: 4.9)
Side of the lesion	7 left, 4 right
Location of the lesions	2 lesions in the WM adjacent to the precentral cortex
	2 lesions in the corona radiata
	2 lesions in the posterior limb of the internal capsule
	2 lesions in the cerebral peduncle
	2 lesions extending from the cerebral peduncle to the internal capsule
	1 lesions extending from the cerebral peduncle to the corona radiata
Time from hemiparesis	Mean: 14 months (SD: 16)

EDSS, Expanded Disability Status Scale; FS, functional system; WM, white matter.

Twelve age- and gender-matched healthy controls [mean age 39.7 years (SD 14.2), six female] were studied.

### Differences between the affected and unaffected side

In patients, there were no significant differences in any of the WM and GM measures between the affected and unaffected side. In controls, there were no differences between the left and right side in any of the measures.

### Differences between groups and association with disability

**Figure 4.**
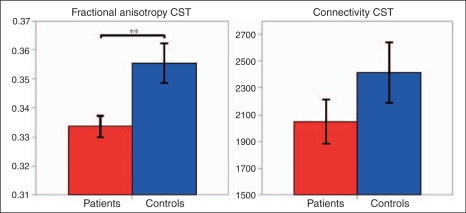
Fractional anisotropy and connectivity of the corticospinal tract (CST) in patients (in red) and healthy controls (in blue). Error bars represent the SEM. **p = 0.007.

**Figure 5.**
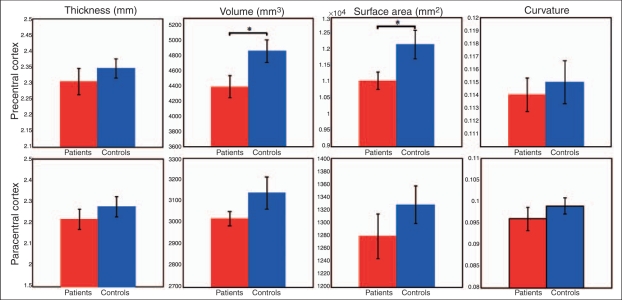
Measures of the precentral and paracentral cortices in patients and in healthy controls. Error bars represent the SEM. *p < 0.05.

Patients had significantly lower CST FA than controls (*p* = 0.007) (Table 2, Figure 4). There was a trend towards lower CST connectivity in patients (*p* = 0.065) (Table 2, Figure 4). Patients had a smaller volume and surface area of the precentral cortex than controls (*p* = 0.028 and *p* = 0.038, respectively) (Table 2, Figure 5). The thickness and curvature of the precentral cortex, as well as the volume, thickness, surface area, and curvature of the paracentral cortex, although lower in patients than in controls, did not reach statistical significance.

**Table 2.** CST and PMC measures in patients and controls

	Patients [mean (SD)]	Healthy Controls [mean (SD)]	*p*-values	Controls-Patients [difference (%)]	95% CI	
					lower	upper
Connectivity	2047.7 (546.3)	2413.5 (812.5)	n.s.^a^	365.8 (17.8%)	−100.9	832.5
FA	0.334 (0.012)	0.355 (0.023)	*p* = 0.007	0.021 (6.3%)	0.059	0.378
Thickness (mm)						
Precentral	2.3054 (0.1369)	2.3445 (0.1076)	n.s.	0.0391 (1.7%)	−0.0609	0.1558
Paracentral	2.2155 (0.1611)	2.2744 (0.1678)	n.s.	0.0589 (2.7%)	−0.0792	0.2108
Volume (mm^3^)						
Precentral	11,045 (915)	12,169 (1580)	*p* = 0.028	1124 (10.2%)	161	2378
Paracentral	3,019 (114)	3,139 (269)	n.s.	120 (4%)	−134	389
Surface area (mm^2^)						
Precentral	4,395 (487.5)	4,866 (542.8)	*p* = 0.038	471 (10.7%)	33	944
Paracentral	1,279 (114.5)	1,328 (106.5)	n.s.	49 (3.8%)	−54	140
Curvature						
Precentral	0.114 (0.0043)	0.115 (0.006)	n.s.	0.001 (0.9%)	−0.0041	0.0047
Paracentral	0.096 (0.009)	0.099 (0.0068)	n.s.	0.003 (3.1%)	−0.0041	0.0102

a *p* = 0.065. CI, confidence interval; CST, corticospinal tract; FA, fractional anisotropy; n.s., not significant; PMC, primary motor cortex.

**Figure 6.**
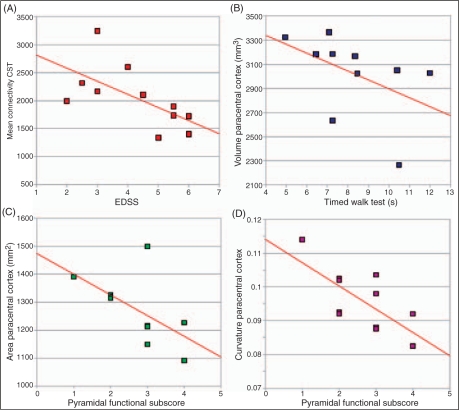
Graphs showing the correlations between **A:** corticospinal tract (CST) connectivity and Expanded Disability Status Scale (EDSS), **B:** volume of the paracentral cortex and timed walk test (TWT), **C:** surface area of the paracentral cortex and pyramidal functional system (FS) subscore, and **D:** curvature of the paracentral cortex and pyramidal FS subscore. Regression lines are shown on each scatter plot.

In patients, there was a negative correlation between CST connectivity and EDSS (rho −0.71, *p* = 0.015) (Figure 6A). Although there is a suggestion that lower CST connectivity correlates with greater pyramidal FS score (rho −0.58, *p* = 0.06), and lower CST FA with greater EDSS (rho −0.47, *p* = 0.074), these relationships were not statistically significant [95% confidence interval (CI): −0.875 to 0.03 and −0.834 to 0.18, correspondingly]. In patients, a smaller volume of the paracentral cortex was associated with an increasing time to complete the TWT (rho −0.71, *p* = 0.022) (Figure 6B). Furthermore, the surface area and the curvature of the paracentral cortex in patients were lower with increasing pyramidal FS score (rho −0.65, *p* = 0.030, and rho −0.63, *p* = 0.037, respectively) (Figure 6C, D). Cortical thickness did not correlate with disability (cortical thickness and EDSS: rho −0.51, *p* = 0.11, 95% CI: −0.849 to 0.129; cortical thickness and TWT: rho −0.56, *p* = 0.093, 95% CI: −0.868 to 0.06; thickness paracentral and EDSS: rho −0.55, *p* = 0.083, 95% CI: −0.864 to 0.074; cortical thickness and TWT: rho −0.54, *p* = 0.093, 95% CI: −0.86 to 0.088).

### Relationship between grey matter and white matter measures

In patients, lower connectivity of the CST affected by the lesion correlated with greater surface area of the ipsilateral paracentral cortex (rho −0.6, *p* = 0.05). This association was also significant with bootstrap analysis. Conversely, lower connectivity in the unaffected CST correlated with lower surface area of the ipsilateral paracentral cortex (rho 0.6, *p* = 0.05). None of the remaining correlations between WM and GM measures were significant.

## Discussion

In this study, we combined two advanced imaging techniques, probabilistic tractography and FreeSurfer cortical surface-based methodology, to perform a comprehensive *in vivo* assessment of the motor system, including the CST and the PMC, and investigate the contribution of WM and GM damage to disability in MS.

With regard to the WM assessment, we found that patients had significantly lower CST FA than controls, but CST connectivity correlated with EDSS and pyramidal FS score better than CST FA. This suggests that connectivity is a measure complementary to FA. These findings extend previous investigations, which have either assessed the FA along the CST^9,44,45^ or tested for correlations between connectivity and disability in MS^4,6^ and ALS.^1,5^ Furthermore, a recent study that specifically investigated the relationship of MRI abnormalities in the CST with lower limb weakness, revealed a moderate but significant quantitative association between disability and tract-specific MRI changes, in keeping with our results.^46^

A novelty of our study is that we obtained several measures of the PMC, other than the volume and tested motor-system specific hypotheses. The most interesting results from these analyses are: (i) patients had lower surface area and volume of the precentral cortex than controls; this finding gives insight into the mechanisms of cortical atrophy in MS, indicating that the loss of volume may occur due to the reduction in the surface area rather than in the thickness; (ii) the surface area and curvature of the paracentral cortex correlated with motor disability, being lower in patients with higher pyramidal FS score; (iii) smaller paracentral cortex volume was associated with worse walking ability, as measured by the TWT. These results suggest that new cortical measures of the PMC, such as surface area and curvature, reflect damage that contributes to functionally relevant impairment. These GM measures should, therefore, be used in future studies to quantify clinically relevant abnormalities in cortical morphology in MS.

The potential relationship of these novel measures with the underlying pathological abnormalities known to occur in MS is interesting, and deserves further studies that compare imaging measures with histological findings. Surface area and curvature may reflect changes in cortical architecture due to either intrinsic GM pathology or WM abnormalities. In terms of GM pathology, neuronal loss is the major determinant of cortical atrophy, while focal cortical demyelination is less relevant.^47^ Importantly, loss of dendritic and axonal projections of the surviving axons also contributes to cortical atrophy.^48^ It is therefore possible that the reduction in surface area detected in patients when compared with controls is driven by the loss of neurons and dendritic arbors, while the corresponding correlation with disability reflects the functional consequences of these pathological processes. Conversely, it seems more likely that cortical curvature is driven by loss of volume in the underlying WM. However, at present, it is not possible to distinguish between these processes using MRI alone, and it is likely that a combination contributes to the observed changes in cortical measures. An important consideration is that a methodological bias during segmentation/parcellation of the cortex may have contributed to the observed changes in cortical surface and curvature between patients and controls and their correlation with disability. For example, one could hypothesize that the automated definition of specific cortical areas, which uses sulcal and gyral anatomy, would be less accurate in patients than controls, if the cortical surface anatomy in patients is grossly abnormal. However, the results of our cortical segmentation were checked visually for each subject, and were considered to be correct and consistent between groups. Furthermore, it should be noted that the FreeSurfer methodology has been validated in previous studies using phantom and post-mortem material,^31–40^ and that the results from a large number of studies using this technique for cortical segmentation *in vivo* in different patient groups, including MS, appears remarkably reproducible.^49–51^

With respect to the PMC thickness, we did not find differences between groups, which may be related to the moderate disability of our patients, compared with a previous study.^14^ However, the patient sample size was small and may well have limited the detection of subtle group differences. On the other hand, the lack of correlation between the PMC thickness and disability is in agreement with another study.^12^ Further studies are needed to clarify the contribution of focal thinning of the PMC to motor disability.

Our investigation of the relationship between CST and PMC measures in patients gave intriguing results. On the affected side, the surface area of the paracentral cortex increased as tract connectivity decreased, while the opposite was true for the unaffected side, where the surface area increased with increasing tract connectivity. This may imply that a lesion in the CST causes significant structural changes in the morphology of the PMC. Functional MRI studies in MS provide evidence for both inter- and intrahemispheric reorganization of PMC activation,^52–54^ and our results possibly reflect the structural correlates of this functional adaptation.^55^ This important issue warrants further investigation: future studies will permit a better understanding of the relationship between (i) the mechanisms of WM and GM damage in specific brain regions and (ii) the mechanisms of functional and structural adaptation in the motor system in MS. In particular, our understanding of the way in which WM pathology drives cortical atrophy and reorganization will benefit greatly from longitudinal studies, which will assess cortical measures, including the newly developed techniques that are introduced here, in relation to functionally relevant WM lesions over time.

Our study has some limitations. First, our sample size was relatively small, although carefully selected to represent patients with a previous episode of hemiparesis. It is possible that the sample size may be too small to detect a true finding, and, therefore, some of our non-significant results could be false negatives. For example, as shown on Table 2, connectivity in the CST of patients decreases by as much as 17.8% when compared to controls, though this remains non-significant; indeed, the 95% CIs associated with this sample difference were quite wide (−100.9, 832.5). Similarly, we cannot exclude the possibility that some of the non-significant correlations of the MRI measures with disability represent false negative results. In particular, in the case of the non-significant correlations between FA and EDSS or thickness of the PMC and both EDSS and TWT, although the magnitude of the coefficient suggests a negative association between FA or cortical thickness and disability, the corresponding 95% CIs were again quite wide. Therefore, in the future, larger studies will be required to confirm the results reported here. Second, a large number of statistical tests (about 90) were performed, without formal correction for multiple comparisons. Nevertheless, for an alpha level of 0.05, one would expect on average 4.5 out of 90 false positive results. However, we reported 10 significant results for a *p*-value of ≤0.05, making a type I error very unlikely to account for all these significant results. Furthermore, this is a hypothesis-driven rather than an exploratory study, making the need for multiple comparison corrections less relevant.^56^

Notwithstanding these limitations, the present study has demonstrated the utility of applying advanced MRI to assess structural damage in the WM and GM of the motor system in MS and provide possible markers of structural damage that is clinically relevant. Imaging functional systems that are important for disability, and, in particular, the assessment of both the WM and GM damage within these systems, may emerge as a reliable method to test specific hypotheses in MS and in other neurological diseases.
